# A Comparison of Functional Outcomes in Rotator Cuff Repairs Using Adjunctive Bone Marrow Aspirate Concentrate vs. Bone Marrow Aspirate Concentrate With Platelet-Rich Plasma: A Systematic Review and Meta-Analysis

**DOI:** 10.7759/cureus.67594

**Published:** 2024-08-23

**Authors:** Seth J Spicer, Sara Soliman, Robert Malek, Mitchell Kaplan, Jensen Clark, Nicholas Averell, Brandon Goodwin, Richard Jermyn

**Affiliations:** 1 School of Medicine, Rowan-Virtua School of Osteopathic Medicine, Stratford, USA; 2 Research, Futures Forward Research Institute, Toms River, USA; 3 Surgery, Rowan-Virtua School of Osteopathic Medicine, Stratford, USA; 4 Internal Medicine, Rowan-Virtua School of Osteopathic Medicine, Stratford, USA; 5 Medicine, Rowan-Virtua School of Osteopathic Medicine, Stratford, USA; 6 Pain Management, Rowan-Virtua School of Osteopathic Medicine, Stratford, USA; 7 Physical Medicine and Rehabilitation, NeuroMusculoskeletal Institute, Rowan-Virtua School of Osteopathic Medicine, Stratford, USA

**Keywords:** shoulder arthroscopy, orthopedic surgery, stem cell therapy, regenerative medicine, platelet rich plasma, bone marrow aspirate concentrate, rotator cuff repair

## Abstract

Regenerative medicine, specifically bone marrow aspirate concentrate (BMAC) and platelet-rich plasma (PRP), has become a novel adjunct that orthopedic surgeons have started to use with surgical rotator cuff repairs (RCR). Thus, we are conducting this systematic review to determine if either RCRs with BMAC alone or with BMAC and PRP result in superior functional outcomes. We conducted a comprehensive search using five databases including PubMed, Web of Science, Embase, Scopus, and Cochrane. After duplicates were removed, 1205 studies were screened by title and abstract using Rayyan, resulting in three included studies (one BMAC with PRP and two BMAC only). Only studies that reported functional outcomes using the American Shoulder and Elbow Surgeons Shoulder Score and the University of California Los Angeles Shoulder Score were included. Changes in assessment scores from baseline to follow-up evaluation were quantified using the effect size and used in the meta-analysis for each group. Interpretation of treatment efficacy was represented using Cohen’s d. The effect size of BMAC with PRP (Cohen’s d = 2.19) was not significantly different (p = 0.76) from that of BMAC alone (Cohen’s d = 2.35). Between-group differences in functional outcomes were Cohen's d = 0.16, which was not significant. Given the lack of superiority and the small sample size, more research is required before a conclusion can be drawn as to the benefits of combining PRP with BMAC for RCR. If functional outcomes are the same, using BMAC alone as an adjunct may be optimal to reduce resources used and cost. Future studies should be conducted with a larger pool as our primary limitation is that only three studies were included.

## Introduction and background

Rotator cuff injuries are the most common shoulder ailment treated by orthopedists, ranging from tendon inflammation to partial and even complete tendon tears [1]. In cadaver dissections, the frequency of these tears as reported in the medical literature ranges from 5-40%, greatly depending on the thickness of the tears and symptomatology [1,2]. Asymptomatic tears, which are often underdiagnosed, are prevalent in almost 30% of patients 60 years of age and older and 62% among patients 80 years of age and older [3]. Diagnosing and addressing rotator cuff tears (RCTs) early on is critical as early detection can prevent progression to symptomatic or even full-thickness tears, which may result in more debilitating symptoms and require more complex and costly treatments. Those with symptomatic RCTs often face limitations with daily activities, such as lack of strength above the shoulder, inability to use their respective arm overhead, nocturnal pain, and even the inability to sleep on the affected side [3,4].

Treatment for RCTs varies significantly depending on the thickness and severity of the tear [3]. However, conventional treatment starts with more conservative options, such as physical therapy for shoulder range of motion and cuff strengthening, non-steroidal anti-inflammatory agents (NSAIDs), and corticosteroid injections [5]. If these treatments are refractory, surgical intervention becomes appropriate [3,4]. Surgical options involve either open or arthroscopic reattachment of the relevant tendon back to its insertion site, either the greater or lesser tuberosity [3,4]. A more radical treatment option for complex cases consists of total shoulder replacement [6]. Unfortunately, after tendon reattachment and recovery from RCTs, re-tear rates range from 13% to 94% in the literature [4,7]. For example, while Denard PJ et al. reported a success rate of 78% in a study involving 126 rotator cuff repairs, Henry P et al. reported a re-tear rate of 79% following massive rotator cuff repairs [6,8]. However, these rates are greatly affected by risk factors such as age, smoking, initial tear size, tendon length, and even repair technique [7,9-11]. Irrespective of the cause, more efficient methods to treat RCTs are critical to reduce healthcare costs. Annually, 250,000 rotator cuff repairs are performed with an average cost ranging from $6,367 to $25,353 per patient [12,13]. Furthermore, studies need to account for the fact that patients are also financially burdened by lost work income due to disability from the initial injury, surgical repair, and missed workdays. This yields the need for finding adjuvant treatments that improve our biology to reduce healthcare costs associated with subsequent operations due to re-tear and prolonged recovery.

The novelty of regenerative medicine is that previously injured and diseased tissue can be self-repaired, negating the need to rely heavily on surgical interventions associated with high re-tear rates [14]. For RCT, common regenerative methods involve injecting platelet-rich plasma (PRP) and bone marrow aspirate concentrate (BMAC) [14]. PRP is an orthobiologic increasingly used in regenerative medicine for the treatment of musculoskeletal conditions, including tendinopathy [15]. PRP is a biological product that has a markedly increased number of platelets in a fraction of plasma. The rationale for its use and the therapeutic potential of high platelet values relies on its capacity to supply extraordinary amounts of growth factors. Growth factors play roles in the migration, differentiation, and proliferation of cells as well as in the biosynthesis of the extracellular matrix, leading to repair and regeneration in tissues that have low healing potential [16].

The efficacy of PRP in muscle and tendon injuries remains uncertain. Martinez-Zapata MJ et al. used a combination of PRP (ed-L-PRP-IIB2) and a rehabilitation program for acute hamstring injuries and concluded that this combination was more effective than rehabilitation alone [17]. In contrast, Hamilton B et al. evaluated the effect of PRP injections (Red-L-PRP-IIB2) in athletes and did not see a change in recovery time [18]. Reurink G et al. had similar results and did not observe a difference in patients receiving intramuscular PRP injections (PRP-IA2) compared to control for hamstring injuries [19]. Specific to tendon healing, a systematic review and meta-analysis of eight papers found that the addition of PRP had no impact on the functional or clinical improvements in Achilles tendinopathy [20]. Further, another systematic review and meta-analysis assessing the effect of PRP on tendinopathy across thirteen randomized controlled trials found no statistically significant differences compared to placebo [21].

Although the use of BMAC is newer, the results for tendon treatment are promising [22]. However, the literature on the use of stem cells for flexor tendon repair in animal models, chronic patellar tendinopathy, and rotator cuff repair presents mixed results [23-25]. While both treatments are gaining interest, the existing research on the combined efficacy of PRP and BMAC for human tendinopathy is nearly non-existent. One meta-analysis of seventeen studies did find decreased re-injury rates in horse tendons when both modalities were used; however, research in humans on the combined efficacy is of great clinical importance [26].

There is growing interest in the field of regenerative medicine and increasing literature on the benefits of concurrent BMAC and PRP, but there is no consensus on the efficacy of their combined utilization. As a result, the authors are conducting this systematic review of current evidence to assess functional outcomes when using BMAC and BMAC with PRP in the management and treatment of RCTs [14].

## Review

Methods

A systematic review and meta-analysis were conducted employing the PRISMA 2020 guidelines [27].

Inclusion Criteria

Studies that consisted of primary clinical data on the use of BMAC augmentation for rotator cuff repair and BMAC with PRP for rotator cuff repair were analyzed. Case-control series, cohort studies, prospective studies, retrospective reviews, and randomized control trials were included in the inclusion criteria. The outcome of interest was post-operative rotator cuff function. Articles were not excluded based on the date of publication, language of publication, or nation of origin.

Exclusion Criteria

Article formats excluded from analysis included articles without full text available, studies still in progress without any reported data, and animal studies. Articles that did not include a bone marrow aspirate concentrate arm or included additional augmentations other than BMAC in their study design were also excluded.

Information Sources and Search Strategy

This systematic review utilized five medical databases to search for articles on the use of bone marrow aspirate concentrate augmentation for rotator cuff repair and BMAC with PRP for rotator cuff repair. These databases included PubMed, Web of Science, Embase, Scopus, and Cochrane Library. Search terms utilized were: (“Platelet-rich plasma” OR “platelet rich plasma” OR “PRP” OR “Bone marrow aspirate concentrate” OR “BMAC” OR “cBMA” OR “bone marrow concentrate” OR “bone marrow stem cells” OR “bone marrow mesenchymal stem cell concentrate” OR “Bone Marrow Mesenchymal Stem Cells” OR “concentrated bone marrow aspirate”) AND (“Rotator cuff repair” OR “RCR” OR “rotator cuff tear” OR “rotator cuff”). The article search was conducted by SS for all literature published before 04/18/2024. Duplicate studies were identified and resolved utilizing Rayyan.ai online software. Following the identification of duplicate articles, two reviewers (SS and RM) manually sorted through the retrieved articles to ensure no further duplicates existed.

Study Selection

**Figure 1:**
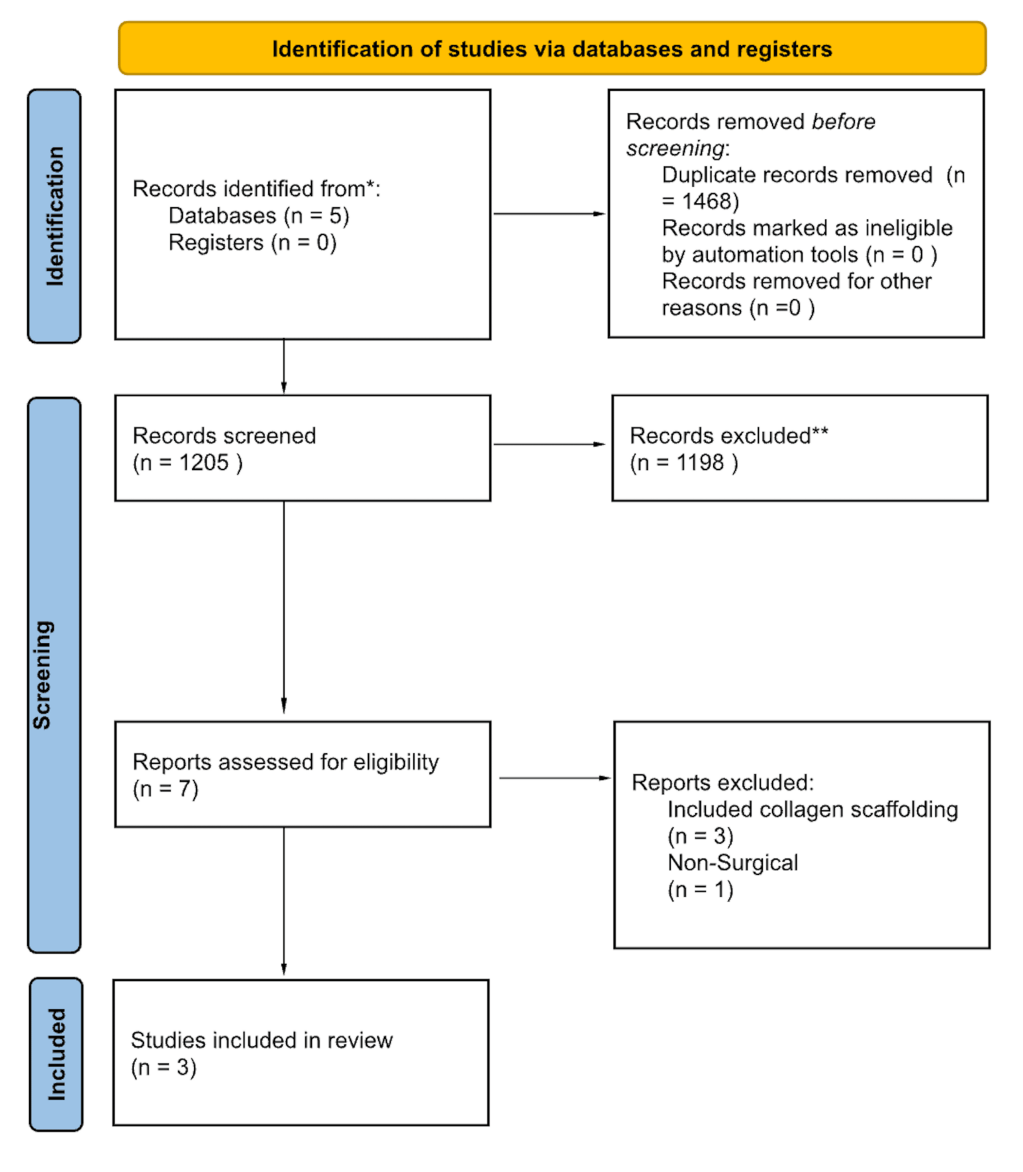
Article selection flow sheet per PRISMA 2020 guidelines; certainty of evidence and risk of bias assessment. *The five databases searched were PubMed, Web of Science, Scopus, Embase, and Cochrane. **Studies were screened independently via title and abstract by two reviewers against the inclusion and exclusion criteria. Included were studies of any language, publication time, or design, that consisted of primary clinical outcome data on the use of BMAC augmentation for rotator cuff repair and BMAC with PRP for rotator cuff repair. Articles were not excluded based on the date of publication, language of publication, or nation of origin. Excluded were articles without full text available, studies still in progress without any reported data, animal studies, those that did not include a bone marrow aspirate concentrate group, and those that included additional augmentations other than BMAC. BMAC: Bone marrow aspirate concentrate.

Once duplicates were identified and sorted through, title and abstract analysis were conducted for inclusion. Following title and abstract analysis, a full-text appraisal was completed by two trained reviewers (SS and RM). Studies determined to be eligible for data analysis (both quantitative and qualitative) were subjected to data extraction. In total, 1468 duplicates were removed, 1205 articles were screened, 7 full texts were assessed, and 3 papers met the inclusion criteria (Figure 1).

Data Collection

The full-text appraisal was performed through an initial critical appraisal, followed by data extraction. Extracted data were examined for relevance, significance, and generalizability. The primary measure the reviewers extracted was rotator cuff function following BMAC augmentation compared to BMAC and PRP augmentation. Studies were then subjected to a critique of their design.

Certainty of Evidence and Risk of Bias Assessment

**Figure 2:**
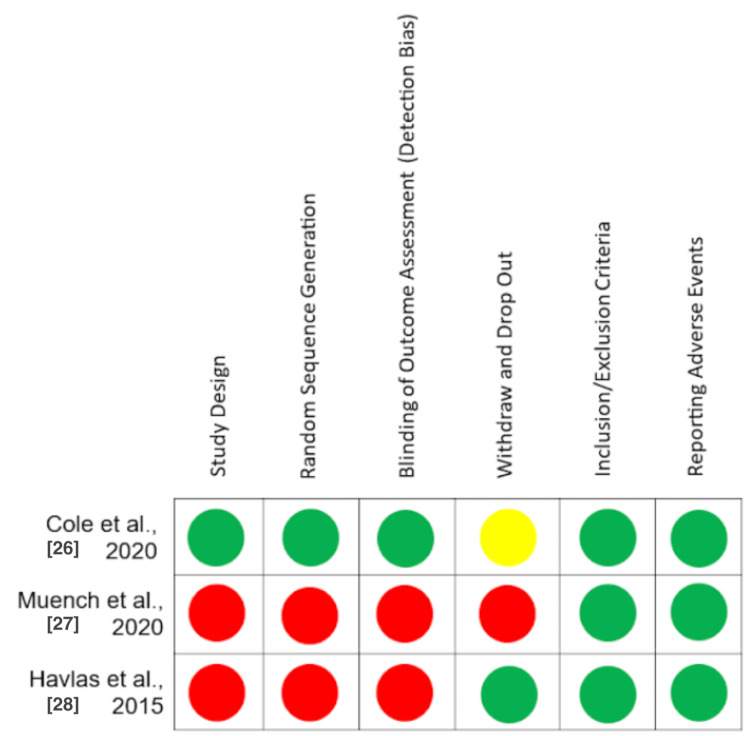
Bias assessment using the ROBINS-I tool. Green = Low risk of bias, Yellow = Moderate risk of bias, Red = High risk of bias.

Included articles were subjected to scoring for certainty of evidence using the Grading of Recommendations Assessment, Development, and Evaluation (GRADE) criteria, independently by two authors (MK and NA). Bias in each article was assessed based on their respective study designs, independently by two authors (MK and NA). Study bias was evaluated using the Newcastle-Ottawa scale and the ROBINS-I tool. Bias varied between studies and is displayed in Figure 2. The GRADE assessment ranged in quality from low to high, resulting in an overall determination of a “low” level of evidence.

Results

Effect of Intervention

The primary variable of interest was patient function scores at baseline compared to follow-up. The average follow-up time was 10.1 months post-intervention, and function was represented by the ASES scores in two studies and the UCLA Scores in one. A comprehensive literature search resulted in three studies consisting of sixty-six participants. There was one study in the BMAC group with sixteen participants and two studies in the BMAC plus PRP group containing fifty participants in total. In all studies included, the participants' functional scores improved significantly when comparing pre-treatment and post-treatment.

**Figure 3:**
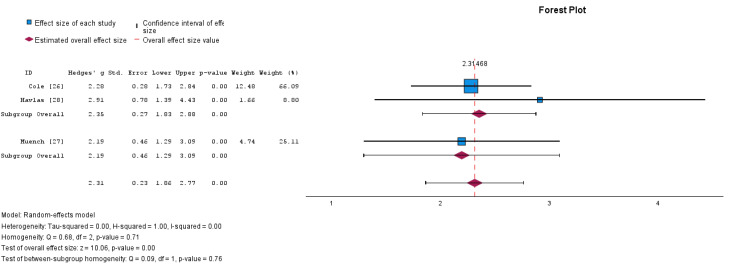
Forest plot of the random-effects model meta-analysis comparing the combination of PRP and BMAC versus BMAC alone in the augmentation of arthroscopic rotator cuff repair. Subgroup 1 is BMAC alone; subgroup 2 is BMAC with PRP. Subgroup and overall effect sizes are provided. BMAC: Bone marrow aspirate concentrate; PRP: Platelet-rich plasma.

To determine the efficacy of this dual treatment modality in improving functional scores, a meta-analysis with subgroup assessment was performed between scores of function pre-treatment and post-treatment. Results were displayed via a forest plot in Figure 3. Each subgroup analysis was significantly different from the baseline (p < 0.01). The effect size for the RCR with BMAC only was Cohen's d = 2.35 (95% CI: 1.86-2.84). The effect size for the RCR with BMAC and PRP adjuncts was Cohen's d = 2.19 (95% CI 1.34 to 3.05). There was a negligible difference between the effect sizes of each subgroup (Cohen's d = 0.16) that was not significantly different as supported by a non-statistically significant test of between subgroup homogeneity (p = 0.76) (Figure 3).

*Heterogeneity* 

The heterogeneity of the three studies was assessed in SPSS and represented by Tau-squared (𝛕2), H2, and I2. Measures of heterogeneity for the BMAC-only group were found to be 𝛕2 = 0.000, H2 = 1.00, and I2 = 0%, while the measures of variance for the BMAC plus PRP group could not be calculated since there was only one study in the group.

Discussion 

In this systematic review, we have critically assessed the utilization of PRP and BMAC as treatment options for rotator cuff tear management, as well as their impact on functional outcomes. We report the most up-to-date systematic review on this topic as of April 2024. There is still no consensus on the clinical superiority of BMAC in addition to PRP as adjuncts for recovery from rotator cuff repairs.

Clinical Efficacy

All of the studies included in our analysis highlighted that both PRP and BMAC had some efficacy as adjuncts to rotator cuff repair in improving patient outcomes. While both therapies were proven to be effective, there was no significant difference in effect between the use of singular vs. combined therapies. Cole BJ et al. showed decreased re-tear rates, however, there was no improvement in treatment failures and the need for additional surgery. The study did not show significant differences between the BMAC group and the control group [28]. Case studies from both Muench LN et al. and Havlas V et al. showed that the use of BMAC and PRP therapies was safe and effective at improving functional outcomes [29,30]. While BMAC and PRP add to the cost, this may result in lower overall healthcare costs due to their ability to improve patient’s functional outcomes, recovery, and re-tear rates.

Variability in Outcomes

Analysis of heterogeneity and homogeneity between subgroup analyses was performed on the studies included in our systematic review. Low scores of heterogeneity and tau-squared highlight the similarities and low variability between the results of the multiple studies. Homogeneity testing of subgroups was not statistically significant; thus, no conclusions could be drawn in favor of PRP in addition to BMAC for RCR due to costs and risk.

In this study, we sought to provide an in-depth review of the existing literature on platelet-rich plasma and bone marrow aspirate concentrate injections for rotator cuff tear management. Our systematic review screened 1,205 articles, however, only three articles met our criteria. Although the quality of studies and evidence varied, it was clear that there were substantial improvements in function following rotator cuff repair for all included studies. The PRP concentration from the Muench study utilized 60ml of whole blood which was reduced to 2-3ml of PRP which was added to BMAC, in contrast to the other studies which only utilized BMAC. Future studies should assess these findings in comparison to RCR without adjunctive therapy.

Limitations and Future Directions

The findings of this systematic review support the potential of BMAC and PRP as strong treatment options for patients with rotator cuff tears. However, there is still a limited amount of high-level evidence in the form of randomized clinical trials. The studies analyzed investigated outcomes in fewer than 100 participants, weakening the potential for generalizability. Since this field of study is relatively new, there are few primary research articles to draw information from. To assess as much of the relevant current literature as possible, a pertinent study with a potential confounding variable was included. The study by Muench LN et al. is a case report on outcomes after rotator cuff repair supplemented with subacromial bursa, BMAC, and PRP, not just BMAC and PRP [30]. Subacromial bursa may contain MSCs conducive to healing [30]. The addition of this factor might have introduced a confounding variable, increasing the likelihood of a false positive. Among the other articles, factors such as tear size, chronicity of the tear, tendon quality, and tendon fixation techniques were either absent or not uniform across the studies analyzed. The addition or absence of these factors may have influenced the outcomes of the respective studies. More randomized clinical trials will help elucidate the role and effectiveness of these regenerative therapies in patient management and are needed to draw replicable conclusions. Going forward, research should be dedicated to analyzing concentration and procedural techniques to contribute to developing a system of standardization. Prior studies showed no differences in cell concentrations between different harvesting techniques; thus, further analysis of the clinical effects of the different techniques used is needed for the development of standardization of the BMAC procedure [31]. PRP research also suffers from a lack of standardization. Currently, there is no agreed-upon standard for the protocol regarding the technique and concentration of PRP injectate. Concentrations often go unreported in studies, making the analysis of efficacy difficult. Researchers must include characteristic analysis of whole blood products and subsequent analysis of PRP products in their study design and report it accordingly. The establishment of standardized criteria and protocols for both PRP and BMAC will allow for more uniformity in future trials, enabling more statistical analysis and conclusions to be drawn.

It would also be beneficial for studies to use scoring systems that are associated with the best validity and reliability. Not all orthopedic surgeons utilize the same scoring system, which makes it difficult to conduct a homogeneous comparison. In a study conducted by Wylie JD, the authors discuss the advantages and disadvantages of each scoring system and outline which are most reliable [32]. Studies like this should be utilized to create an overall consensus among orthopedic surgeons about which scoring systems should be utilized to make studies more homogeneous and comparable.

## Conclusions

Both BMAC and PRP have become useful adjuncts used by orthopedic surgeons for RCR. However, a current challenge is delineating which combination of therapies will yield the most optimal results in terms of functional outcomes. In our limited analysis using the UCLA Shoulder Score and ASES scores as outcome measures, we found that using BMAC alone is equally effective as using BMAC with PRP. This study calls for additional research to be conducted to validate the results of this review. Additionally, future primary studies should also explore additional parameters, such as cost, overall patient satisfaction, number of injections needed, operative time, and retear rates.
